# Personalized Sudden Cardiac Death Risk Stratification in Hypertrophic Cardiomyopathy: Beyond Conventional Risk Scores

**DOI:** 10.3390/jpm16060287

**Published:** 2026-05-26

**Authors:** Jacopo Costantino, Federico Ballatore, Daniele Porcelli, Barbara Romani, Massimiliano Campoli, Lorenzo Maria Zuccaro, Giulia Marchionni, Maria Alfarano, Samuel Costantino, Cristina Chimenti

**Affiliations:** 1 Department of Medical and Cardiovascular Sciences, Sapienza University of Rome, 00185 Roma, Italy; federico.ballatore@uniroma1.it (F.B.); m.afarano@policlinicoumberto1.it (M.A.); costantino.1884105@studenti.uniroma1.it (S.C.); cristina.chimenti@uniroma1.it (C.C.); 2 Department of Medicine, University of Padua, 35128 Padova, Italy; 3 Department of Cardiology, San Pietro Fatebenefratelli Hospital, 00189 Rome, Italy; daniele.porcelli@hotmail.com (D.P.); barbararomani75@gmail.com (B.R.); campoilimassimiliano@tiscali.it (M.C.); lorenzomzuccaro@gmail.com (L.M.Z.)

**Keywords:** hypertrophic cardiomyopathy, sudden cardiac death, implantable cardioverter-defibrillator, risk stratification, late gadolinium enhancement, mavacamten

## Abstract

Hypertrophic Cardiomyopathy (HCM) is one of the most common inherited cardiomyopathies and remains an important cause of ventricular arrhythmias and sudden cardiac death (SCD), particularly in younger individuals. Although the annual incidence of arrhythmic death is relatively low in contemporary cohorts, identifying those patients who may benefit from primary prevention with an implantable cardioverter-defibrillator (ICD) remains a major clinical challenge. Current risk stratification strategies rely on two principal paradigms. The European approach is centered on the HCM Risk-SCD score, whereas the American approach is mainly based on major clinical risk markers. Both strategies have important strengths and limitations, reflecting the persistent difficulty of accurately predicting arrhythmic events in such a heterogeneous disease. The HCM Risk-SCD score has demonstrated robust external validation and high specificity for identifying patients at higher risk, but it may fail to recognize some vulnerable individuals who remain below conventional treatment thresholds. For this reason, several additional risk modifiers have gained increasing relevance in contemporary practice. Among them, extensive late gadolinium enhancement, left ventricular systolic dysfunction, apical aneurysm, and clinically meaningful genetic findings may provide important incremental prognostic information beyond traditional models. Emerging disease-modifying therapies, in particular Mavacamten, may also influence future risk assessment. However, whether these improvements translate into a true reduction in SCD risk remains uncertain. Importantly, the decision to implant an ICD should not depend on numerical risk alone. It should arise from a process of shared decision-making integrating estimated risk, treatment burden, competing comorbidities, age, lifestyle, and patient values. In this context, the concept of an individualized threshold of “acceptable risk” becomes central. In conclusion, prevention of SCD in HCM is moving beyond conventional scores toward a personalized and dynamic framework in which predictive tools, advanced phenotyping, evolving therapies, clinical expertise, and patient preferences are combined to guide individualized care.

## 1. Introduction

Hypertrophic Cardiomyopathy (HCM) is one of the most common inherited cardiomyopathies in the general population, with an estimated prevalence of approximately 1:200–1:500 individuals [1]. It is characterized by otherwise unexplained left ventricular hypertrophy, defined as an increase in myocardial wall thickness that cannot be solely accounted for by abnormal loading conditions or by another cardiac, systemic, or metabolic disease capable of producing the observed magnitude of hypertrophy [2].

HCM is most frequently caused by pathogenic variants in genes encoding sarcomeric proteins and is commonly inherited in an autosomal dominant pattern with variable penetrance and expressivity [3]. However, a positive family history is not required for diagnosis, as de novo variants and clinically silent familial disease may occur. The disease affects both sexes, although women are often diagnosed later and may present with more advanced symptoms at the time of recognition [4].

In adult patients, the clinical diagnosis of HCM is generally established by cardiac imaging demonstrating a maximal end-diastolic wall thickness ≥ 15 mm in one or more left ventricular myocardial segments, in the absence of another cause of hypertrophy [5,6]. Lesser degrees of hypertrophy (13–14 mm) may also be considered diagnostic in first-degree relatives of patients with HCM or in individuals carrying a pathogenic or likely pathogenic variant associated with the disease [5,6].

The clinical course of HCM is highly heterogeneous, ranging from asymptomatic individuals with normal life expectancy to patients who develop major complications such as heart failure, atrial fibrillation, progressive functional limitation, thromboembolism, and ventricular arrhythmias leading to sudden cardiac death (SCD), the most devastating complication in HCM [1,2]. Although the annual incidence of arrhythmic death in contemporary HCM cohorts is relatively low—generally estimated at <1% per year—the absolute burden remains clinically relevant because of the relatively high prevalence of the disease [7]. Moreover, HCM has historically been recognized as one of the leading causes of SCD in young individuals and competitive athletes, particularly in North America, with younger patients generally exhibiting a higher arrhythmic risk than older adults [4].

Accordingly, one of the major challenges in contemporary HCM management is to identify, among the broad and heterogeneous HCM population, those individuals at sufficiently high risk to benefit from primary prevention with an implantable defibrillator (ICD). This task remains complex, as arrhythmic risk is dynamic, multifactorial, and only partially captured by conventional prediction models.

The aim of this narrative review, primarily focused on contemporary ESC and ACC/AHA guideline frameworks and complemented by key studies addressing established and emerging modifiers of SCD risk in HCM, is not only to summarize the established and emerging determinants of SCD risk in adult HCM but also to discuss how contemporary SCD prevention in HCM is progressively evolving from a score-based model toward a personalized, patient-centered, and dynamic framework integrating clinical judgment, advanced phenotyping, evolving therapies, and patient preferences.

## 2. Defining the Threshold of “Acceptable Risk”

A central concept in the prevention of SCD in patients with HCM is that risk stratification cannot be separated from the consequences of the preventive therapy itself. The main strategy currently available for primary prevention is implantation of an ICD. While device technology continues to evolve—with improved algorithms, longer battery longevity, enhanced sensing capabilities, and less invasive options such as subcutaneous systems—ICD therapy remains associated with relevant complications and substantial life implications that should not be underestimated.

Procedure-related complications include pocket hematoma, infection, lead dislodgement, cardiac perforation, and pericardial effusion [8]. During long-term follow-up, patients may also experience device-related infection including endocarditis, tricuspid valve dysfunction, lead failure, generator recalls, need for repeated replacements, and inappropriate shocks. Among these, inappropriate therapies and transvenous lead complications remain particularly important because of their potential impact on morbidity, quality of life, and healthcare burden [8].

These issues are especially relevant in younger individuals, who are more likely to live for decades with the device, undergo multiple generator changes, and accumulate a greater lifetime probability of complications or interventions. Accordingly, the balance between expected benefit and treatment burden is often more complex in younger patients than in older individuals with shorter projected exposure time.

Beyond medical complications, ICD implantation may influence several domains of daily life. These include professional activities, driving eligibility, participation in sports, body image perception, anxiety related to shocks, device awareness, family dynamics, and broader psychological, social, or cultural factors. Importantly, the relative weight of these factors is highly variable and deeply individual, meaning that the same estimated arrhythmic risk may be perceived very differently by different patients.

Comorbidities and life expectancy must also be integrated into decision-making. In patients with advanced non-cardiac disease, frailty, or limited survival expectancy, the absolute benefit of primary prevention may be substantially reduced despite the presence of conventional risk markers.

This leads to the key concept of “acceptable risk.” The clinician’s role is first to define, as accurately and evidence-based as possible, the patient’s estimated arrhythmic risk through comprehensive evaluation. However, the decision to implant an ICD should not rely on risk estimation alone. Rather, it emerges from the intersection between quantified risk and the individual patient’s values, age, comorbidity profile, lifestyle, and preferences. Consequently, the threshold at which risk becomes “high enough” to justify intervention is not universal, but personalized.

Importantly, this patient-centered perspective is strongly endorsed by contemporary guidelines. Both the European Society of Cardiology and the American College of Cardiology/American Heart Association recommendations emphasize that decisions regarding primary prevention ICD implantation should arise from a process of shared decision-making, in which estimated arrhythmic risk is discussed alongside procedural risks, long-term consequences, competing comorbidities, and the patient’s informed preferences and goals [5,6].

## 3. Two Different Perspectives: United States vs. Europe

Over the last two decades, substantial efforts have been made to improve risk stratification for SCD in patients with HCM, leading to the development of two principal decision-making frameworks for primary prevention with an ICD.

**Figure 1:**
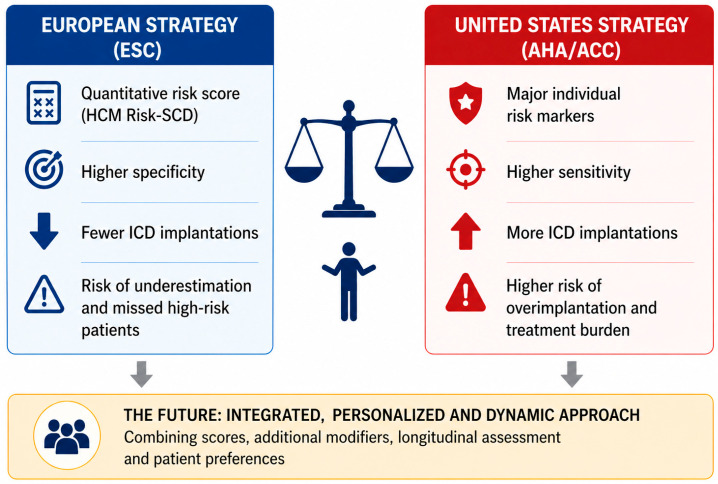
Comparison Between the European and American Risk Stratification Strategies.

The contemporary European and American approaches reflect two partially different philosophies (Figure 1). The European Society of Cardiology guidelines recommend the use of the HCM Risk-SCD model, a multivariable tool that estimates 5-year SCD risk using a combination of weighted clinical and imaging variables, including age, family history of SCD, unexplained syncope, non-sustained ventricular tachycardia (NSVT), maximal left ventricular wall thickness, left ventricular outflow tract gradient, and left atrial diameter [9]. This model provides an individualized quantitative estimate intended to support risk discussion and ICD selection.

By contrast, the American College of Cardiology/American Heart Association strategy is primarily based on the presence of major risk markers rather than on a single numerical score [6]. In this framework, ICD implantation is considered when one or more high-risk features are present, including unexplained syncope, family history of HCM-related SCD, NSVT, apical aneurysm, extensive fibrosis, massive hypertrophy, or left ventricular systolic dysfunction.

These heterogeneous approaches underscore a fundamental reality: no currently available model fully captures the complexity of arrhythmic risk in HCM. They also reflect different priorities, balancing sensitivity, specificity, prevention of avoidable deaths, exposure to device-related complications, and healthcare resource utilization [10,11,12].

A long-standing debate remains as to which strategy should be preferred. Broadly speaking, the American approach has traditionally prioritized broader identification of potentially at risk of arrhythmic events to minimize the risk of leaving potentially vulnerable patients without ICD protection, whereas the European model has generally aimed to improve treatment selectivity and reduce unnecessary implantations and long-term device burden [12]. Importantly, these differences should not be interpreted as rigidly opposing strategies, as clinical decision-making is also influenced by age, clinical context, center expertise, and shared decision-making.

An important real-world perspective comes from the Sarcomeric Human Cardiomyopathy Registry (SHaRe), an international collaborative dataset including major centers from both North America and Europe. Comparative analyses suggest that ICD implantation rates in the United States are substantially higher than in Europe, approximately twofold in some cohorts (HR 2.27 [1.89–2.74]). In parallel, American patients receiving ICDs often present with fewer conventional risk markers than their European counterparts, indicating a lower threshold for preventive implantation [13].

Interestingly, although ICD implantation rates were substantially higher in the United States, rates of appropriate ICD therapies have been reported to be higher in European cohorts (US vs. non-US sites HR 0.52 [0.28–0.97]), whereas overall SCD outcomes appear broadly similar between the two regions (HR 1.21 [0.74–1.97]) [13]. Taken together, these observations may suggest that a more selective strategy can improve treatment efficiency without compromising survival. However, such findings should be interpreted with caution, as differences in referral patterns, patient selection, follow-up duration, and local practice may significantly influence outcomes.

Rather than framing the comparison in terms of a superior or inferior strategy, the experiences from Europe and the United States highlight the complementary strengths and limitations of the two paradigms. A more sensitive approach may reduce missed events, whereas a more specific strategy may limit unnecessary implants and treatment burden.

**Figure 2:**
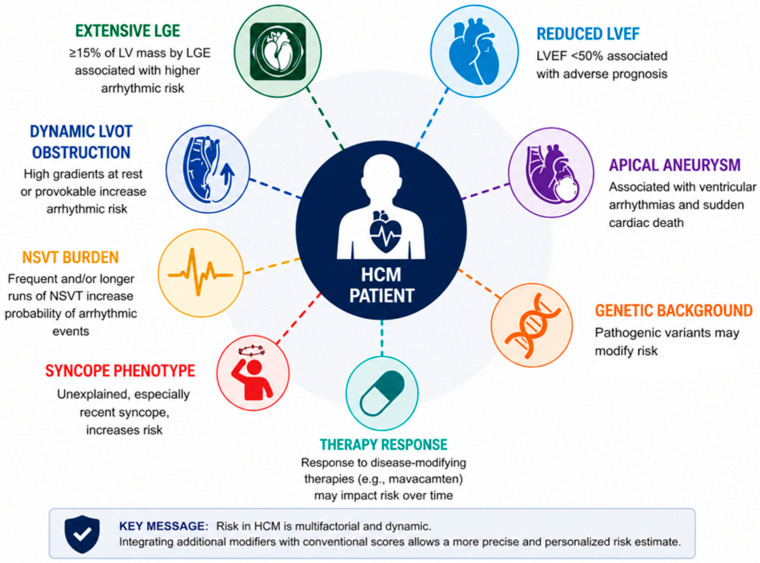
Risk Factors for sudden cardiac death in Hypertrophic Cardiomyopathy.

From a practical standpoint, a reasonable contemporary approach may be to use structured tools such as the HCM Risk-SCD score as an initial framework to identify clearly high-risk patients, and then to refine risk assessment in apparently low- or intermediate-risk individuals through additional modifiers, as discussed in the following sections (Figure 2).

## 4. HCM Risk-SCD Score: Strengths, Limitations, and Clinical Interpretation

Among the currently available tools for arrhythmic risk stratification in HCM, the HCM Risk-SCD score remains the most widely adopted multivariable model in contemporary practice, particularly in Europe [9,14,15,16]. Developed by the European Society of Cardiology, it was designed to provide an individualized estimate of 5-year risk of sudden cardiac death and to support decisions regarding primary prevention with an ICD [5]. The model is based on seven routinely available clinical and imaging variables derived from large observational cohorts: age, family history of SCD, unexplained syncope, non-sustained ventricular tachycardia (NSVT), maximal left ventricular wall thickness, left ventricular outflow tract (LVOT) gradient, and left atrial diameter [9]. Unlike binary risk-marker approaches, these variables are not assigned equal importance but are weighted according to their independent prognostic contribution within the original regression model, thereby generating a continuous patient-specific estimate rather than a simple categorical classification. According to ESC recommendations, patients are generally categorized into three groups based on predicted 5-year SCD risk: low risk (<4%), in whom ICD implantation is usually not recommended; intermediate risk (4–6%), in whom ICD implantation may be considered; and high risk (≥6%), in whom ICD implantation should be considered after comprehensive clinical evaluation [5].

These thresholds were introduced to facilitate decision-making, while recognizing that no universally accepted absolute risk threshold mandates device therapy and that clinical judgment remains essential in borderline scenarios. Over time, the HCM Risk-SCD score has demonstrated robust clinical utility and has been externally validated in multiple independent studies across different healthcare systems and patient populations [14,15,16]. Its relevance has become such that even the 2024 American College of Cardiology guidelines on HCM, historically centered on a major risk-marker strategy, acknowledged the score for the first time as a useful element within the shared decision-making process [6].

One of its main strengths is its relatively high specificity in identifying patients at the highest arrhythmic risk, thereby helping to reduce unnecessary ICD implantations and the long-term burden associated with device therapy [12]. However, the score is less effective in identifying all patients who may still experience malignant ventricular arrhythmias despite remaining below conventional treatment thresholds [11]. Within the broad population classified as low or intermediate risk, there may still be individuals harboring substantial arrhythmic vulnerability who would benefit from protection [11]. This limitation reflects, at least in part, the complexity of HCM and the imperfect nature of some variables included in the model. A major example is unexplained syncope, which carries substantial weight in the score. Although recent unexplained syncope is associated with increased arrhythmic risk, not all syncopal episodes in HCM are caused by ventricular arrhythmias [17,18,19]. Reflex-mediated syncope, vasovagal episodes, orthostatic hypotension, situational triggers, transient hemodynamic compromise, or dynamic obstruction may produce a similar presentation. In everyday practice, distinguishing arrhythmic from non-arrhythmic syncope is often difficult and frequently relies on retrospective patient history, which may be incomplete or inaccurate. Similarly, the prognostic role of NSVT is well recognized, but its detection is strongly dependent on the duration and intensity of rhythm monitoring [20]. A patient undergoing standard 24 h Holter monitor surveillance may show no arrhythmia, whereas the same individual could have NSVT documented during prolonged external monitoring, wearable technologies, or an Implantable Loop Recorder [21]. In this sense, NSVT detection is partly stochastic: the longer the monitoring window, the greater the probability of capturing transient arrhythmic episodes. This creates an important interpretative challenge, as the original score was developed in an era of shorter monitoring strategies, and it remains uncertain how episodes detected through contemporary long-term surveillance should be weighted today. Furthermore, another element of uncertainty concerns the very definition of NSVT itself. Although NSVT is generally defined as a run of consecutive ventricular beats lasting from 3 beats up to 30 s, different studies and guideline documents have not always adopted uniform criteria regarding episode duration, ventricular rate required for an event to be classified as NSVT potentially contributing to variability in its reported prognostic significance [14].

Additional limitations concern structural variables including potential variability in maximal wall thickness measurements between echocardiography and cardiac magnetic resonance imaging. In patients with extreme hypertrophy, particularly when maximal wall thickness exceeds 30–35 mm, the relationship between wall thickness and arrhythmic risk may not be linear, leading to potential underestimation of risk in a subgroup historically considered particularly vulnerable [22,23]. Likewise, the inclusion of the LVOT gradient may be problematic, as this parameter is highly dynamic and influenced by loading conditions, exercise, posture, hydration status, and medical therapy. Moreover, its association with SCD is less consistent than with symptoms or progression to heart failure, making its specific contribution to arrhythmic prediction more controversial. Furthermore, although the HCM Risk-SCD model has demonstrated important external its calibration and prognostic performance may be less consistent in specific subgroups that were underrepresented in the original derivation cohorts (pediatric populations, genotype-positive phenotype-negative individuals, patients with extensive LGE or apical aneurysm, and those undergoing contemporary disease-modifying therapies).

Taken together, these limitations do not diminish the importance of the HCM Risk-SCD score, but rather define its correct role in modern practice. The score should not be interpreted as a definitive answer, but as a structured and evidence-based starting point. To achieve truly personalized prevention of SCD in HCM, numerical risk estimates must be integrated with additional markers, advanced imaging, genotype, longitudinal reassessment, and individualized clinical judgment.

## 5. Additional Risk Modifiers Beyond the Score (Table S1)

### 5.1. Apical Aneurysm

An apical aneurysm is identified in approximately 2–5% of patients with HCM represents a distinct phenotypic subset characterized by localized thinning and dyskinesia of the left ventricular apex, often surrounded by areas of fibrosis and scar [24,25]. The association between apical aneurysm and ventricular arrhythmias was first reported more than three decades ago and has subsequently been confirmed by multiple observational studies [26,27,28]. More recent evidence has also suggested that aneurysm size may carry incremental prognostic relevance, with larger aneurysms potentially associated with greater arrhythmic risk [29].

These findings progressively influenced guideline recommendations. As previously discussed, the American College of Cardiology/American Heart Association guidelines consider apical aneurysm a major risk modifier supporting a Class IIa indication for primary prevention with an ICD [6]. Similarly, prior European guidelines on ventricular arrhythmias and SCD prevention also recognized its potential relevance [30].

However, more recent analyses have introduced a more nuanced interpretation. Apical aneurysm is frequently observed in patients with more advanced or adverse HCM phenotypes, often characterized by greater scar burden, more severe remodeling, systolic impairment, and higher baseline HCM Risk-SCD estimates [24,31]. This raises the possibility that the excess arrhythmic risk associated with apical aneurysm may not derive exclusively from the aneurysm itself as an independent arrhythmogenic substrate, but rather from the broader disease severity of the patients in whom it occurs [31]. In this perspective, the aneurysm may function more as a marker of advanced disease than as an isolated causal risk factor.

This evolving concept has been partly reflected in more recent European recommendations. The 2023 European Society of Cardiology guidelines on cardiomyopathies adopted a more cautious position, suggesting that the presence of apical aneurysm should be interpreted within the overall risk profile rather than automatically prompting a change in strategy beyond standard multivariable risk assessment [5].

From a practical standpoint, apical aneurysm remains an important red flag that should trigger careful evaluation, particularly when associated with scar, systolic dysfunction, documented ventricular arrhythmias, or progressive remodeling. Whether it should be considered an independent indication for ICD implantation in all patients, however, remains an area of ongoing debate and exemplifies the broader need to integrate phenotype-specific features with formal risk models rather than relying on any single marker in isolation.

### 5.2. Late Gadolinium Enhancement

One of the most frequently cited limitations of the HCM Risk-SCD score is the absence of late gadolinium enhancement (LGE) assessment by cardiac magnetic resonance (CMR). This criticism is understandable, as the original model was developed during a period when Cardiac Magnetic Resonance Imaging was less widely available and evidence regarding the prognostic significance of myocardial fibrosis was still emerging.

Today, LGE is recognized as one of the most relevant imaging markers in the contemporary management of HCM. Multiple studies have shown that the presence of LGE is associated with a higher risk of adverse outcomes, including all-cause mortality, cardiovascular mortality, ventricular arrhythmias, and SCD [32,33,34,35]. Accordingly, any modern strategy for arrhythmic risk stratification cannot ignore this parameter.

However, the interpretation of LGE is more complex than a simple present-versus-absent variable. LGE is fundamentally a quantitative marker, and the key clinical question is not merely whether fibrosis exists, but how much fibrosis is present and at what threshold arrhythmic risk becomes meaningfully increased. This has important implications for decision-making, particularly when considering primary prevention with an ICD.

A further source of heterogeneity is the lack of a universally accepted method for LGE quantification. Different techniques—including manual planimetry, signal-threshold approaches, and the commonly used 6 standard deviation (6-SD) method—may generate substantially different estimates of fibrosis burden. As a consequence, direct comparison across studies is not always straightforward, and reported cut-offs may vary depending on the methodology used [36].

Overall, when focusing on the most robust available evidence, the 6-SD method appears to be one of the most clinically applicable approaches, and an LGE extent of approximately 15% of left ventricular mass has emerged as a meaningful threshold associated with increased arrhythmic risk [37]. This value was notably incorporated by the 2023 European Society of Cardiology cardiomyopathy guidelines to define extensive LGE [5].

Beyond total burden, recent investigations have explored whether additional characteristics of fibrosis—such as regional distribution, transmurality, entropy, or progression over time—may further refine risk prediction [38,39,40]. Although these concepts are biologically plausible and potentially promising, current evidence remains less mature and not yet sufficiently standardized for routine clinical implementation.

In practical terms, LGE should be viewed as a central component of personalized risk assessment in HCM. Rather than replacing established clinical models, it provides incremental information that may be particularly valuable in patients with borderline conventional risk estimates, helping to identify individuals in whom the arrhythmic substrate may be more advanced than suggested by clinical variables alone.

### 5.3. Left Ventricular Systolic Dysfunction

Left ventricular systolic dysfunction represents another clinically relevant factor that should be considered beyond the traditional HCM Risk-SCD score [5,6]. Although only a minority of patients with HCM develop overt impairment of systolic function, reduction in left ventricular ejection fraction (LVEF) is widely recognized as a marker of advanced disease and adverse remodeling [41].

Patients with reduced LVEF have consistently been shown to carry a higher risk of major outcomes, including all-cause mortality, heart failure progression, cardiovascular hospitalization, ventricular arrhythmias, and SCD [41,42,43,44,45]. For this reason, regardless of the calculated HCM Risk-SCD estimate, systolic dysfunction cannot be overlooked when evaluating arrhythmic risk in clinical practice.

From a pathophysiological perspective, the transition from the classic hyperdynamic phenotype toward systolic impairment often reflects progressive myocardial fibrosis, chamber remodeling, energetic failure, and increasing electrical instability [45]. In this context, reduced LVEF may be interpreted not only as a marker of pump dysfunction, but also as a surrogate of a more advanced arrhythmogenic substrate.

This concept is reflected in contemporary guideline recommendations. The 2024 American College of Cardiology/American Heart Association guidelines consider LVEF < 50% a major risk factor, supporting consideration of primary prevention with an ICD within a framework of clinical judgment and shared decision-making [6].

The 2023 European Society of Cardiology cardiomyopathy guidelines adopt a more cautious position, acknowledging that the additional and independent prognostic value of systolic dysfunction beyond existing multivariable risk tools is less clearly established [5]. This more conservative interpretation likely reflects the difficulty of distinguishing whether reduced LVEF is an autonomous arrhythmic predictor or rather a marker of globally advanced disease.

In practical terms, however, the presence of systolic dysfunction should always prompt heightened attention. Even when not formally integrated into all risk calculators, reduced LVEF identifies a subgroup of patients with more severe phenotypic progression in whom arrhythmic risk may be underestimated by conventional models alone.

### 5.4. Abnormal Blood Pressure Response to Exercise

An abnormal blood pressure response to exercise is one of the most debated parameters in the field of arrhythmic risk stratification in HCM. Historically, it has been considered a potentially relevant marker, and earlier European recommendations on SCD included it among the additional factors that could raise clinical concern [30]. It is generally defined as either a failure to increase systolic blood pressure by at least 20 mmHg from rest to peak exercise or a fall of more than 20 mmHg from peak exercise pressure [5].

However, the real prognostic significance of this finding remains uncertain. One important limitation is that the studies on which this concept was originally based were highly heterogeneous, with substantial differences in exercise protocols, patient selection, age distribution, and even in the definition of what constituted an abnormal response [46,47,48,49]. This heterogeneity has made it difficult to translate the parameter into a robust and reproducible clinical risk marker.

From a pathophysiological perspective, approximately one-third of adult patients with HCM may exhibit an abnormal systolic blood pressure response during exercise, typically characterized by progressive hypotension or failure to augment systolic pressure appropriately [50]. This phenomenon is thought to result from an inappropriate fall in systemic vascular resistance combined with limited cardiac output reserve. While biologically relevant, this hemodynamic behavior does not necessarily imply the presence of a specific arrhythmogenic substrate.

Indeed, subsequent evidence has substantially tempered the initial enthusiasm surrounding this marker. Although an abnormal exercise blood pressure response may be associated with a higher risk of adverse outcomes in younger patients, particularly those aged ≤ 40 years, its positive predictive value for SCD is low [51,52]. Moreover, in patients older than 40 years, its prognostic significance for ventricular arrhythmias and sudden death appears uncertain. More recent data suggest that, while it may be associated with increased overall mortality—largely driven by heart failure progression—it is not consistently linked to a higher risk of malignant ventricular arrhythmias or SCD [51].

Taken together, these observations suggest that an abnormal blood pressure response to exercise should be interpreted with caution. Rather than being considered a strong stand-alone arrhythmic risk factor, it may be better viewed as a marker of impaired hemodynamic adaptation and, in some cases, of more advanced functional limitation. Its role in contemporary SCD stratification therefore appears more modest than originally proposed, especially when compared with more robust markers such as extensive fibrosis, systolic dysfunction, or apical aneurysm.

### 5.5. Genetics

Another highly debated issue is the role of genetics in arrhythmic risk stratification for HCM. The American and European approaches have not been entirely aligned on this point [5,6]. The 2024 American College of Cardiology/American Heart Association HCM guideline does not include genotype itself among the major stand-alone markers driving the primary prevention ICD algorithm, whereas the 2022 European Society of Cardiology ventricular arrhythmia/SCD guideline had considered the presence of a pathogenic sarcomeric mutation as an additional modifier in selected circumstances [6,30]. In contrast, the 2023 ESC cardiomyopathy guideline moved to a more cautious position and no longer treats sarcomeric positivity, in itself, as a decisive modifier beyond multiparametric clinical assessment [5].

This evolution is understandable. A pathogenic or likely pathogenic sarcomeric variant can be identified in a substantial proportion of patients with HCM, broadly around 40–60% in contemporary series, so a purely dichotomous interpretation—mutation present versus absent—has limited discriminatory power in everyday practice [53]. At the same time, several studies and meta-analyses have shown that sarcomere-positive patients, as a group, have worse clinical profiles and poorer outcomes, including a higher burden of ventricular arrhythmias and sudden death [53,54].

However, this association should be interpreted carefully. Sarcomere-positive patients often show a more severe phenotype overall, with earlier disease onset, more marked hypertrophy, more adverse remodeling, and greater fibrosis burden on cardiac magnetic resonance [25]. Therefore, the currently available evidence does not clearly demonstrate that sarcomeric positivity, considered only as a yes/no variable, is by itself an independent predictor of sudden cardiac death over and above the phenotype it helps shape. In other words, genotype may be linked to higher risk, but often through its association with a more aggressive form of disease rather than as a universally independent risk factor [54].

This is probably where a major methodological problem lies: genetics is too often simplified into a binary variable. Such an approach is reductive. It is increasingly clear that risk is not the same across all sarcomeric genes, nor across all variants within the same gene. Specific mutations in MYH7, for example, have long been associated with more severe phenotypes, while certain troponin-related variants may confer disproportionate arrhythmic risk even when hypertrophy is not particularly extreme [55,56,57]. Multiple or compound sarcomeric mutations may also identify patients with especially adverse outcomes [58].

Accordingly, in a modern personalized approach to SCD prevention, genetic information should not be ignored, but neither should it be interpreted simplistically. The clinically relevant question is no longer whether a patient is merely “genotype-positive,” but which gene is involved, which specific variant is present, how robust its pathogenicity is, and how that genotype interacts with the patient’s phenotype, fibrosis burden, family history, and clinical course. Viewed in this way, genetics is not yet a universal stand-alone indication for ICD implantation, but it remains an important layer of individualized risk interpretation that may meaningfully refine decision-making in selected patients.

### 5.6. Mavacamten: Potential Implications and Current Uncertainties

Another contemporary factor that deserves consideration is the potential impact of disease-modifying therapy on arrhythmic risk assessment, particularly with the introduction of Mavacamten. Recently approved for the treatment of symptomatic obstructive HCM, Mavacamten has consistently demonstrated the ability to markedly reduce the left ventricular outflow tract gradient, thereby substantially relieving dynamic obstruction and improving symptoms, functional capacity, and quality of life [59,60].

Beyond hemodynamic improvement, emerging evidence suggests that mavacamten may induce a broader pattern of positive reverse remodeling. Early studies have reported favorable electrocardiographic and structural changes, including regression of left ventricular wall thickness, reduction in left atrial size, and improvement in markers of myocardial stress [61,62,63,64]. These observations are particularly intriguing because several of these parameters—such as maximal wall thickness, left atrial diameter, and left ventricular outflow tract gradient—are directly incorporated into conventional risk models such as the HCM Risk-SCD score.

Accordingly, treatment with Mavacamten may be associated with a significant reduction in the calculated risk score over time. This raises an important and clinically relevant question: Does improvement in the numerical score truly reflect a parallel reduction in the risk of SCD, or does it simply modify surrogate variables without fully altering the underlying arrhythmogenic substrate? [65].

At present, the answer remains uncertain. Although the structural and functional effects of mavacamten are highly promising, direct evidence demonstrating a reduction in hard arrhythmic endpoints or SCD is still lacking. Therefore, changes in risk estimates observed during therapy should be interpreted cautiously and should not yet be considered equivalent to proven arrhythmic risk reduction.

Nevertheless, mavacamten introduces an important conceptual shift in the field: risk stratification in HCM may no longer be viewed as purely static, but as a dynamic process potentially modified by targeted therapy. Future studies will need to determine whether pharmacologically induced reverse remodeling can translate into meaningful changes in long-term arrhythmic outcomes and ICD decision-making.

## 6. Conclusions

Risk stratification for ventricular arrhythmias and SCD in HCM is complex, multidimensional, and dynamic. No single score or isolated marker can fully capture the biological and clinical heterogeneity of the disease or define, by itself, the optimal threshold for preventive intervention.

Assessment should begin with structured tools such as the HCM Risk-SCD score and then be refined through additional modifiers including LGE extent, left ventricular systolic dysfunction, phenotype-specific features, and clinically meaningful genetic data.

Once patient-specific risk has been defined as accurately as possible, the decision regarding primary prevention ICD implantation depends on whether that risk lies above or below the individual threshold of acceptable risk. This threshold varies over time and across patients according to age, comorbidities, lifestyle, and preferences.

In this evolving landscape, the future of SCD prevention in HCM lies not in a single perfect model, but in a personalized approach combining phenotype characterization, disease evolution, therapeutic response, clinical expertise, and patient values.

## Data Availability

No new data were created or analyzed in this study. Data sharing is not applicable to this article.

## Supplementary Materials

The following supporting information can be downloaded at: https://www.mdpi.com/article/10.3390/jpm16060287/s1, Table S1: Conventional Risk Variables, Additional Modifiers, and Clinical Caveats in Contemporary SCD Risk Stratification for HCM.

## Abbreviations

The following abbreviations are used in this manuscript:

ACC	American College of Cardiology
AHA	American Heart Association
CMR	Cardiac Magnetic Resonance
ESC	European Society of Cardiology
HCM	Hypertrophic Cardiomyopathy
HF	Heart Failure
HR	Hazard Ratio
ICD	Implantable Cardioverter-Defibrillator
LA	Left Atrium/Left Atrial
LGE	Late Gadolinium Enhancement
LV	Left Ventricle/Left Ventricular
LVEF	Left Ventricular Ejection Fraction
LVOT	Left Ventricular Outflow Tract
LVOTO	Left Ventricular Outflow Tract Obstruction
NSVT	Non-Sustained Ventricular Tachycardia
ROC	Receiver Operating Characteristic
SCD	Sudden Cardiac Death
SD	Standard Deviation
SHaRe	Sarcomeric Human Cardiomyopathy Registry
VF	Ventricular Fibrillation
VT	Ventricular Tachycardia
