# Supplementary material for: Personalized Sudden Cardiac Death Risk Stratification in Hypertrophic Cardiomyopathy: Beyond Conventional Risk Scores
Source: J Pers Med. 2026 May 26;16(6):287. doi: 10.3390/jpm16060287 (PMC13301690; doi:10.3390/jpm16060287)
Supplement: Supplementary file 1 [file jpm-16-00287-s001.zip › jpm-4312978-supplementary.pdf]

**Supplementary Table S1. Conventional Risk Variables, Additional Modifiers, and Clinical Caveats in Contemporary SCD Risk Stratification for HCM**

| Risk Factor / Modifier | ACC/AHA Approach | ESC Approach | Main Clinical Caveats |
|---|---|---|---|
| Family history of SCD | Major risk marker | Included in score | Variable definition across studies |
| Unexplained syncope | Major risk marker | Included in HCM score | Difficult distinction between arrhythmic and non-arrhythmic syncope |
| NSVT | Major risk marker | Included in HCM score | Strongly influenced by monitoring duration, rate, and arrhythmic burden |
| Maximal wall thickness | Major risk marker (massive hypertrophy) | Included in HCM score | Echo vs CMR variability; non-linear risk at extreme hypertrophy |
| LVOT gradient | Contextual role | Included in HCM score | Dynamic parameter influenced by loading conditions and therapy |
| Left atrial diameter | Limited role | Included in HCM score | Surrogate marker of chronic hemodynamic burden |
| Extensive LGE | Important modifier | Important modifier | Lack of universally standardized quantification methods |
| Apical aneurysm | Major modifier | Context-dependent modifier | May reflect advanced phenotype rather than isolated substrate |
| LVEF <50% | Major risk marker | Additional modifier | Marker of advanced remodeling and systolic dysfunction |
| Genetic background | Limited role | Limited role | Heterogeneous prognostic significance across genes and variants |
| Abnormal blood pressure response to exercise | Limited role | Limited role | Weak specificity for arrhythmic events, especially in adults |
| Mavacamten-related remodeling | Not included | Not included | No direct evidence of reduction in SCD or ventricular arrhythmias |
| Shared decision-making / acceptable risk | Strongly emphasized | Strongly emphasized | Individual thresholds vary according to age, lifestyle, and comorbidities |
